# Mechanical Versus Laser Debridement of SLA Titanium Implants: An In Vitro Morphological and Elemental Analysis of Debris Removal and Surface Preservation

**DOI:** 10.3390/nano16120703

**Published:** 2026-06-06

**Authors:** Baran Yurdakul, Sumeyye Meyvaci, Gokce Aykol-Sahin, Aslan Gokbuget, Funda Yalcin, Ulku Baser

**Affiliations:** 1 Institute of Graduate Studies in Health Sciences, Istanbul University, 34126 Istanbul, Türkiye; baranyurdakul@hotmail.com; 2 Department of Periodontology, Faculty of Dentistry, Istanbul University, 34116 Istanbul, Türkiye; sumeyyemeyvaci@gmail.com (S.M.); fyalcin@istanbul.edu.tr (F.Y.); 3 Department of Periodontology, Faculty of Dentistry, Istanbul Okan University, 34959 Istanbul, Türkiye; gokceaykol@gmail.com; 4 PGG Private Practice, 34365 Istanbul, Türkiye; aslangokbuget@gmail.com

**Keywords:** peri-implantitis, debridement, in vitro, scanning electron microscopy, energy-dispersive X-ray spectrometry, titanium brush, erbium laser, ultrasonic scaler

## Abstract

Peri-implantitis treatment is challenging because of the complex micro- and nanostructured topography of implant surfaces. No standard debridement protocol exists. In this study, we compared five debridement methods used on heavily contaminated titanium implants that were explanted due to peri-implantitis. Twenty-five explanted implants (five per group) were treated with a carbon fiber ultrasonic insert, a polyetheretherketone (PEEK) ultrasonic insert, a rotating titanium brush, an erbium, chromium-doped yttrium, scandium, gallium, and garnet (Er,Cr:YSGG) laser, or an erbium-doped yttrium, aluminum, and garnet (Er:YAG) laser. Five pristine implants were used as controls. Surface morphology was assessed by scanning electron microscopy (SEM). The Modified-Implant Debridement Visual Index (M-IDVI) was used to assess the debridement effectiveness according to SEM images. Surface elemental composition was assessed for atomic percentage (at. %) of carbon, titanium, oxygen and nitrogen using energy-dispersive X-ray spectroscopy (EDS). Mechanical methods were more effective at removing debris than laser methods. The titanium brush showed the lowest residual debris (2.33 ± 0.33) and the greatest reduction in surface carbon (Δ = −7.77 at. %). Surface titanium increased after debridement for all methods except for Er,Cr:YSGG (Δ = −5.9 at. %). Er:YAG best preserved SLA microtopography but exhibited a lower debridement efficacy (3.27 ± 0.83) than mechanical methods. No method resulted in a pristine surface.

## 1. Introduction

Today, implant failures and their associated complications constitute not only an individual issue but also a significant global health burden. Recent systematic reviews and meta-analyses indicate that peri-implantitis affects approximately 18–23% of patients with dental implants worldwide, and between 12% and 20% of implants, depending on the case definition applied [1,2,3,4]. This substantial clinical burden has prompted extensive research into debridement strategies that can re-establish conditions favorable for peri-implant tissue healing [5].

The primary therapeutic goal in peri-implantitis treatment is the thorough removal of surface deposits from the implant surface to enable re-osseointegration [6]. Unlike root surface debridement in periodontitis, the microstructured and threaded geometry of modern dental implants creates protected undercuts and porosities that may act as shelters for bacterial colonization, thus significantly complicating effective mechanical debridement [6,7]. Residual organic contamination, most consistently reflected as increased surface carbon content in spectroscopic analyses, has been shown to impair osteoblast attachment, proliferation, and mineralization on titanium surfaces, with carbon reduction restoring these biological responses [8,9]. Thus, the removal of carbon from the titanium surface has become a central benchmark for evaluating debridement efficacy.

Despite two decades of research, a standardized debridement protocol is yet to be established. The 2023 European Federation of Periodontology S3-level clinical practice guideline and the 2025 Academy of Osseointegration/American Academy of Periodontology (AO/AAP) joint consensus report both acknowledge that no single debridement method has demonstrated definitive superiority, and that the complex surface topography of contemporary implants remains a central therapeutic challenge [10,11]. The companion AO/AAP systematic review on biofilm and surface deposits removal concluded that although mechanical, chemical, laser, and electrolytic modalities can reduce surface contamination, none can reliably restore the elemental profile of a pristine implant surface [12].

Among the available options, erbium lasers (Er:YAG, 2940 nm; Er,Cr:YSGG, 2780 nm) have attracted interest due to their strong absorption by water, which produces an ablation effect with minimal thermal damage to titanium [13,14]. Published laser parameters vary substantially across studies in terms of pulse energy, pulse duration, application time, and handpiece geometry, and this methodological heterogeneity has been identified as a principal barrier to the emergence of a consensus protocol [15]. A previous in vitro study compared three pulse modes of the Er:YAG laser, two energy settings of the Er,Cr:YSGG laser, a titanium curette and a polyetheretherketone (PEEK)-insert ultrasonic scaler on peri-implantitis-failed implants of the same model, and reported that long-pulse Er:YAG produced the closest topography to pristine implant surfaces [16]. Mechanical decontamination devices have undergone parallel evolution, with recent additions including a carbon fiber-reinforced ultrasonic insert designed to minimize surface alteration [17], a PEEK ultrasonic insert introduced as an alternative to a metal insert for implant surface debridement [18,19], and rotating titanium brushes designed to mechanically disrupt surface deposits on the threaded implant surface and supported by both in vitro and clinical evidence [20,21].

The aim of the present in vitro study was to comparatively evaluate the debridement efficacy of contemporary mechanical and erbium laser methods on titanium implants extracted due to peri-implantitis in the context of the broader therapeutic goal of supporting re-osseointegration. The primary outcome was to assess implant surface morphology by scanning electron microscopy (SEM), evaluating the effectiveness of debris removal. Preservation of the SLA microtopography was evaluated separately. The secondary outcome was the change in surface elemental composition measured by energy-dispersive X-ray spectroscopy (EDS). Our null hypothesis was that all debridement methods would produce similar results.

## 2. Materials and Methods

### 2.1. Study Design and Sample

**Figure 1:**
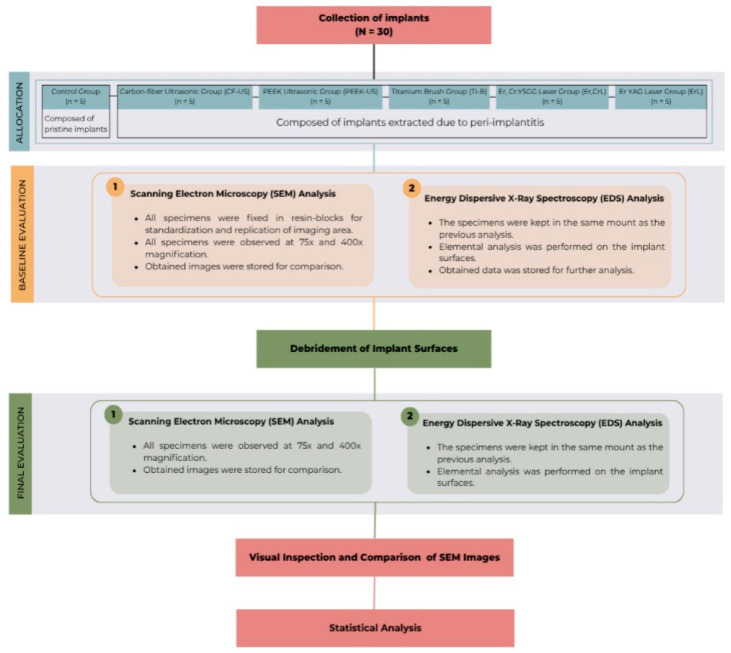
Flowchart of the study design.

In this in vitro study, we evaluated five debridement methods applied to dental implants that failed due to peri-implantitis, and pristine implants were used as the controls. A total of 30 DE2 implants (Detech, Ankara, Türkiye) with a sandblasted, large-grit, acid-etched (SLA) surface and Grade 4 titanium composition were collected. Twenty-five of these implants had been previously explanted due to peri-implantitis and were autoclaved prior to inclusion in the study; five unused implants served as the control group. For the explanted implants to be included in this study, we selected specimens with clearly visible extensive surface deposits. Implants were randomly allocated to five debridement groups (*n* = 5 per group) such that an equal distribution of implants with the same diameter and length was achieved across the groups. All debridement procedures were performed by a single operator (B.Y.) under 2.5× surgical loupe magnification until no visible deposits remained on the surface. Debridement was performed under magnification and standardized conditions until the implant surface appeared visibly clean. An overview of the study design is shown in Figure 1. Ethical approval was obtained from the Istanbul University Faculty of Dentistry Clinical Research Ethics Committee (Approval Number: 2024/77Rev-1).

### 2.2. Specimen Fixation

Each implant was embedded to a depth of 3 mm from the apex in an epoxy-resin block to ensure spatially reproducible imaging and debridement. Control implants were mounted directly on the SEM stub using conductive double-sided tape without resin embedding. Baseline (T0) SEM and EDS measurements were obtained before debridement, and post-debridement (T1) measurements were performed two weeks later. The same protocol was used for each debridement group.

### 2.3. Debridement Procedures

The manufacturer’s recommendations were followed during all interventions. Debridement protocols are shown in Table 1.

#### 2.3.1. Carbon Fiber Insert-Ultrasonic Scaler (CF-US Group)

The Periimpla Soft carbon fiber-reinforced insert was used with the Vector Paro ultrasonic device (Dürr Dental, Bietigheim-Bissingen, Germany) at 23 kHz, maximum water irrigation, and a 30° tip-to-surface angle. The median duration of the debridement was 107 s (s).

#### 2.3.2. PEEK Insert-Ultrasonic Scaler (PEEK-US Group)

The IC1 PEEK insert with an ICS holder was mounted on the MyLunos Duo device (Dürr Dental, Bietigheim-Bissingen, Germany) and operated at 36 kHz, maximum water irrigation, and a 30° tip-to-surface angle. The median duration of the debridement was 124 s.

#### 2.3.3. Rotating Titanium Brush (TiB Group)

The R-Brush titanium brush (NeoBiotech, Seoul, Republic of Korea) was mounted on an X-Cube physiodispenser (Saeshin, Daegu, Republic of Korea) and operated at 4900 rpm under sterile saline irrigation in a clockwise vertical motion. This speed is within the manufacturer-recommended range of 2000–5000 rpm. The median duration of the debridement was 74 s.

#### 2.3.4. Er,Cr:YSGG Laser (ErCrL Group)

The WaterLase iPlus device (Biolase, Foothill Ranch, CA, USA) was used with the RFPT5-14 mm radial-firing periodontal tip (500 µm fiber, 580 µm outer diameter). The settings were as follows: 1.5 W, 30 Hz, 50 mJ/pulse, air 60, water 50, energy density 19.23 J/cm^2^, applied in non-contact mode with a longitudinal sweeping motion. The median duration of the debridement was 332 s.

#### 2.3.5. Er:YAG Laser (ErL Group)

The Fidelis Plus II device (Fotona, Ljubljana, Slovenia) was used with the R02-C handpiece (0.9 mm tip, 0.63 mm^2^ spot area). The settings were as follows: VSP (very short pulse, 100 µs), 100 mJ/pulse, 10 Hz, air 13, water 25, energy density 15.87 J/cm^2^, applied in non-contact mode with a longitudinal sweeping motion. The median duration of the debridement was 114 s.

#### 2.3.6. Control Group

Five pristine implants were examined via SEM and EDS under identical acquisition conditions without any debridement intervention.

### 2.4. SEM Imaging

All SEM images were acquired using a FEI Versa 3D FIB-SEM instrument (Thermo Fisher Scientific, Hillsboro, OR, USA) operating in high-vacuum mode with the Everhart-Thornley detector at 20 kV accelerating voltage and 60 pA beam current. Due to the conductive nature of titanium, specimens were not sputter-coated. Overview images were obtained at 75× magnification to visualize the thread and valley geometry, and detailed images at 400× magnification were obtained from the most heavily contaminated thread of each specimen to examine residual debris and surface damage. A resin-block reference system was used to ensure the same area was captured at T0 and T1.

The Implant Debridement Visual Index (IDVI) was modified for the visual evaluation of SEM images [16]. The Modified-Implant Debridement Visual Index (M-IDVI) aims to evaluate debridement effectiveness by comparing treated, heavily contaminated implants to virgin implants at 75× magnification. The M-IDVI scores are defined as follows:

Score 1—Clean Surface

The implant surface appears completely clean, with no visible residual deposits or contamination. The original surface microtopography is fully preserved.

Score 2—Minimal Residual Contamination

Very small and isolated remnants of debris are visible. The implant surface morphology remains clearly identifiable.

Score 3—Mild Residual Contamination

Limited areas of residual deposits are present, covering less than one-third of the observed surface. Surface characteristics are still predominantly visible.

Score 4—Moderate Residual Contamination

Noticeable debris and contamination are present on one-third to two-thirds of the surface. Surface microtopography is partially obscured.

Score 5—Heavy Residual Contamination

Extensive residual deposits cover more than two-thirds of the surface. Original implant surface features are difficult to distinguish.

Score 6—Severe Contamination

The implant surface is almost completely covered by dense deposits and contaminants, with complete or near-complete masking of the original surface morphology.

### 2.5. EDS Analysis

Elemental analysis was performed using an EDAX Octane Super SDD detector (EDAX LLC., Mahwah, NJ, USA) with EDAX TEAM software version 4.5. (eZAF Smart Quant routine; resolution 128.6–131.2 eV). Titanium, carbon, oxygen and nitrogen atomic percentages were obtained at T0 and T1 for each debridement group and at a single time point for the control group.

### 2.6. Statistical Analysis

A power analysis based on the study by Secgin-Atar et al. [16] detected a difference between debridement methods in the morphologic features of implant surfaces, with a Type 1 error of 5%, a Type 2 error of 20% corresponding to 80% power, and sample size of 5 implants per intervention group. Statistical analyses were performed with IBM SPSS Statistics version 31 (IBM Corp., Armonk, NY, USA). The normality of the data distribution was assessed using the Shapiro–Wilk test. For variables that met normality assumptions, between-group comparisons were performed using one-way ANOVA with post hoc Tukey correction; within-group comparisons were performed using a paired *t*-test with Bonferroni correction. For variables that did not meet normality assumptions, between-group comparisons were performed using a Kruskal–Wallis H test with Bonferroni correction; within-group comparisons were performed using a Wilcoxon-paired test with Bonferroni correction. Statistical significance was set at *p* < 0.05. Inter-rater reliability among the three blinded observers was assessed using the intraclass correlation coefficient (ICC, two-way random-effects model, average measures, absolute agreement).

## 3. Results

### 3.1. Implant Properties and Debridement Durations

The length and diameter of the implants used in the study and their distribution are shown in Table 2. Debridement durations among groups are shown in Table 3 (*p* > 0.05). The ErCrL group had the longest application time, whereas the TiB group was completed in the shortest time. The CF-US group took 107 s, the PEEK-US group took 124 s, and the ErL group took 114 s.

### 3.2. SEM Analysis

SEM analyses were obtained from pre- and post-debridement images of the samples. Three independent blind observers scored the post-debridement images of 25 samples based on M-IDVI (Table 4). The inter-rater reliability was excellent (ICC(2,k) = 0.914). Between-group comparison showed no statistically significant difference (*p* = 0.098) and no pairwise comparisons reached significance after Bonferroni correction (all adjusted *p* > 0.05).

#### 3.2.1. Control Group

**Figure 2:**
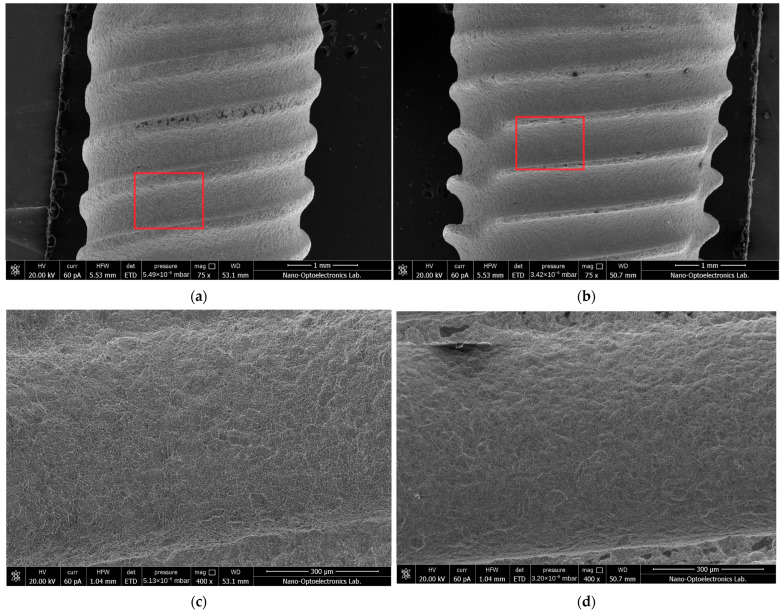
SEM images of pristine SLA titanium control implants. Specimen 1 (**a**,**c**) and Specimen 2 (**b**,**d**). Low-magnification SEM images at 75× (**a**,**b**) with red boxes indicating the high-magnification regions imaged at 400× and corresponding high-magnification images (**c**,**d**) showing the preserved pitted SLA microtopography.

Pristine implants displayed the characteristic SLA microtopography across the entire surface. At 75× (Figure 2a,b) the thread geometry, crest, and valley were sharply defined, and at 400× (Figure 2c,d) the typical pitted, honeycomb-like nanostructure of SLA titanium was uniformly preserved with no visible debris, discoloration, or surface defects.

#### 3.2.2. CF-US Group

**Figure 3:**
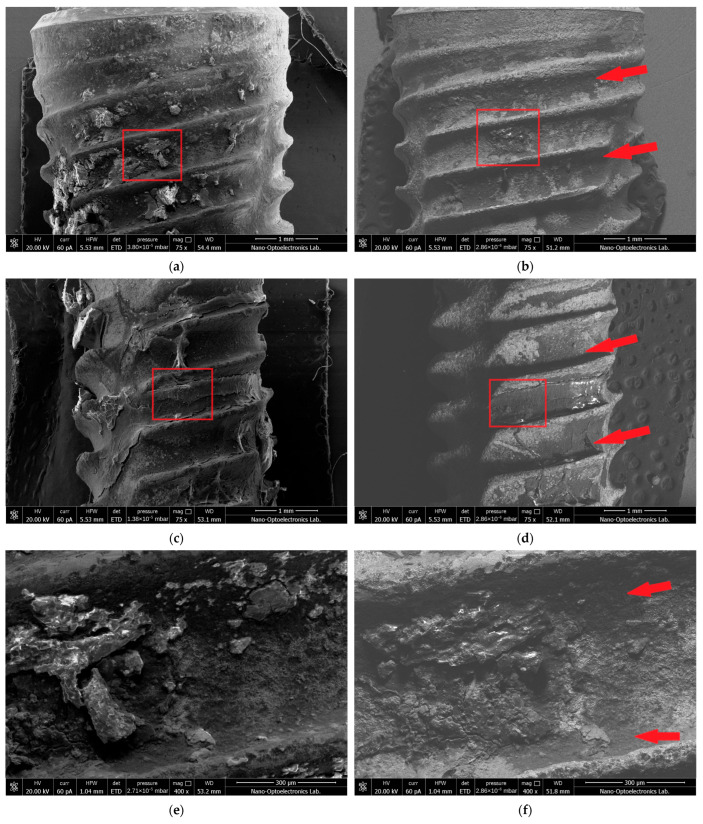

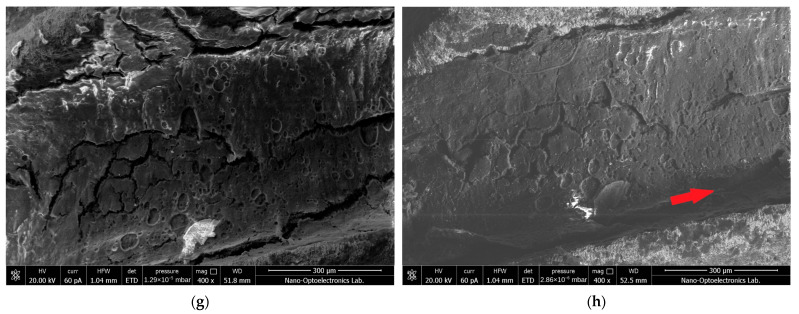
SEM images of CF-US group implants before (T0) and after (T1) debridement: Specimen 3 (**a**,**b**,**e**,**f**); Specimen 4 (**c**,**d**,**g**,**h**). A 75× overview (**a**–**d**) with red boxes indicating the 400× (**e**–**h**) imaging regions; corresponding 400× detail. T0 (**a**,**c**,**e**,**g**) & T1 (**b**,**d**,**f**,**h**). Arrows indicate dark flattened zones within debrided thread areas.

Pre-debridement SEM images at 75× showed widespread debris deposits over the threads and valleys in all five specimens. SEM images of two samples with deposits at T0 were demonstrated (Figure 3a,c,e,g). Post-debridement images showed a visible reduction in surface contamination (Figure 3b,d,f,h). Thread crests and valley geometry became clearly visible, and the thick baseline debris layers were largely removed. Dark and flattened zones (shown with arrows in Figure 3b,d,f,h) were observed within the debrided thread areas, clearly identifiable at 75× and more apparent at 400×. At 400×, the most contaminated valleys showed at least a 50% reduction in surface debris.

#### 3.2.3. PEEK Group

**Figure 4:**
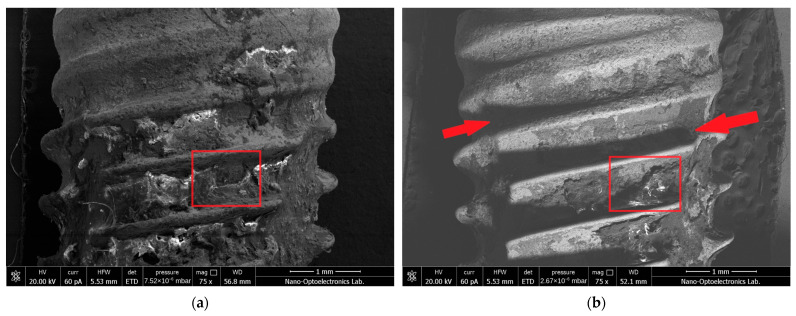

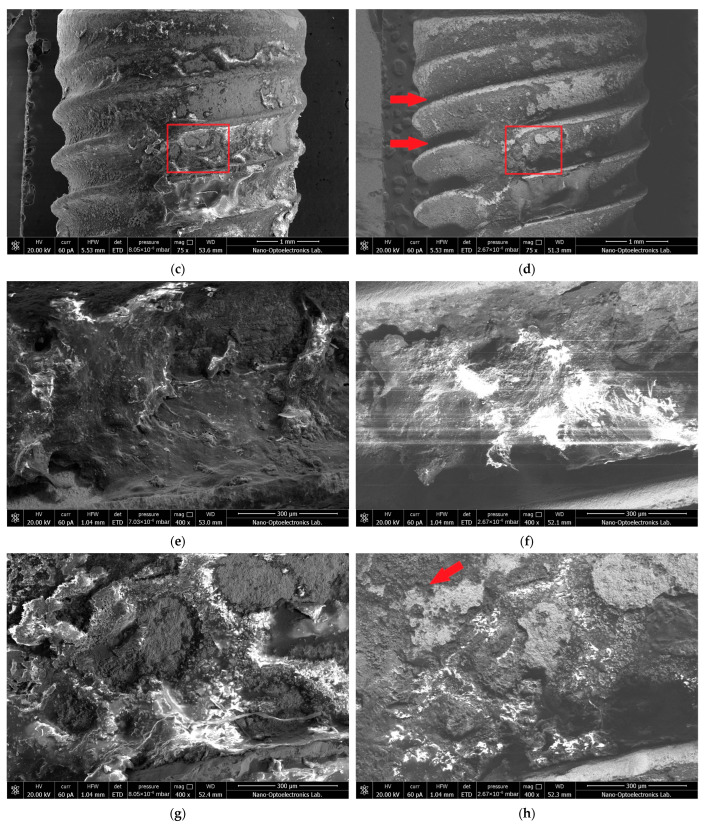
SEM images of PEEK-US group implants before (T0) and after (T1) debridement, Specimen 5 (**a**,**b**,**e**,**f**); Specimen 6 (**c**,**d**,**g**,**h**). A 75× overview (**a**–**d**) with red boxes indicating the 400× (**e**–**h**) imaging regions; corresponding 400× detail. T0 (**a**,**c**,**e**,**g**) & T1 (**b**,**d**,**f**,**h**). Arrows indicate dark patchy zones within debrided regions.

Pre-debridement SEM images at 75× showed extensive debris deposits across the threads and valleys in all five specimens of the PEEK-US group. (Figure 4a,c). Post-debridement images at 75× (Figure 4b,d) showed extensive removal of surface contamination. At 400× magnification, the same regions showed dense debris deposits at T0 (Figure 4e,g), and residual debris remained visible within the thread valleys at T1 (Figure 4f,h). Dark patchy zones (shown with arrows in Figure 4b,d,h) were detectable in the debrided regions. At 400×, the most heavily contaminated valleys showed little visible change between pre- and post-debridement images.

#### 3.2.4. TiB Group

**Figure 5:**
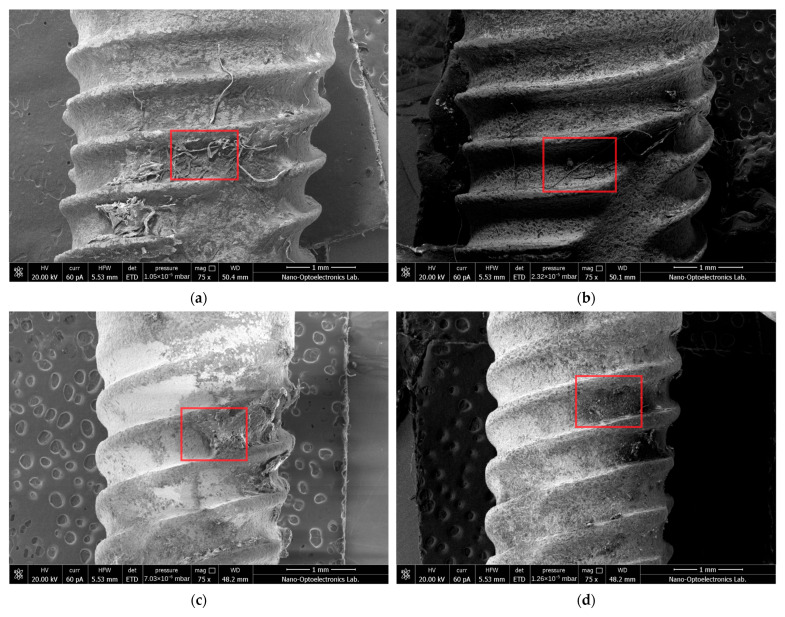

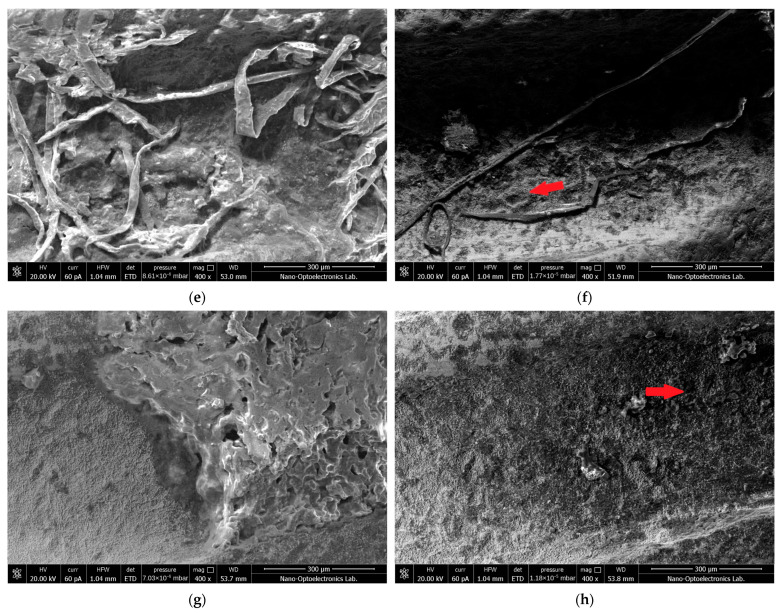
SEM images of TiB group implants before (T0) and after (T1) debridement, Specimen 7 (**a**,**b**,**e**,**f**); Specimen 8 (**c**,**d**,**g**,**h**). A 75× overview (**a**–**d**) with red boxes indicating the 400× (**e**–**h**) imaging regions; corresponding 400× detail. T0 (**a**,**c**,**e**,**g**) and T1 (**b**,**d**,**f**,**h**). Arrows indicate shallow crater-like depressions across the treated surface.

Pre-debridement SEM images at 75× showed extensive surface debris deposits across the threads and valleys in all five specimens of the TiB group (Figure 5a,c). Post-debridement images at 75× (Figure 5b,d) showed that the majority of surface deposits had been removed, with thread crests and valleys becoming sharply visible. At 400× magnification, the same regions showed dense debris deposits at T0 (Figure 5e,g). At T1, the thread-valley junctions had been efficiently cleaned, while shallow crater-like depressions were also visible across the treated surface (arrows in Figure 5f,h).

#### 3.2.5. ErCrL Group

**Figure 6:**
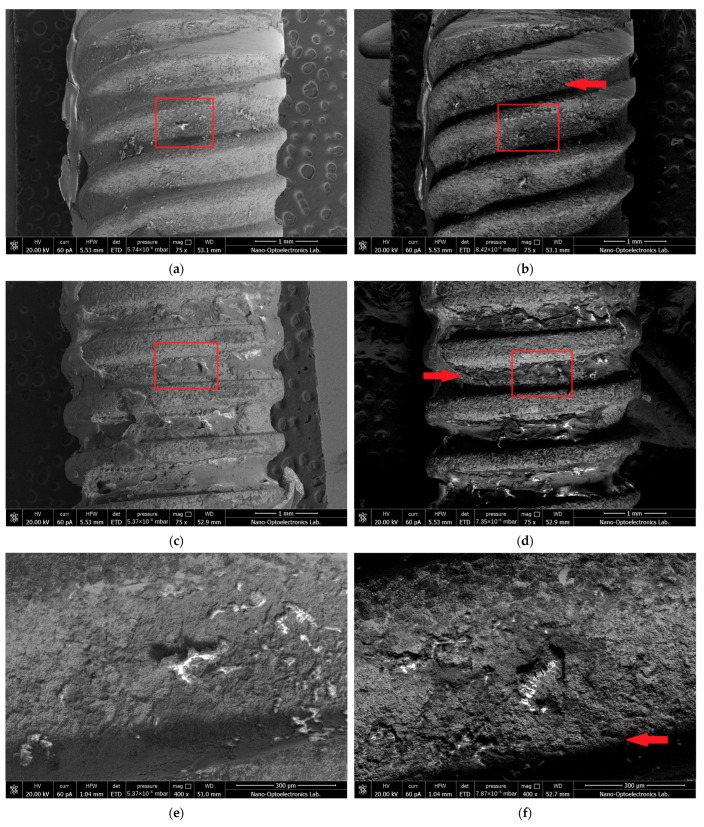

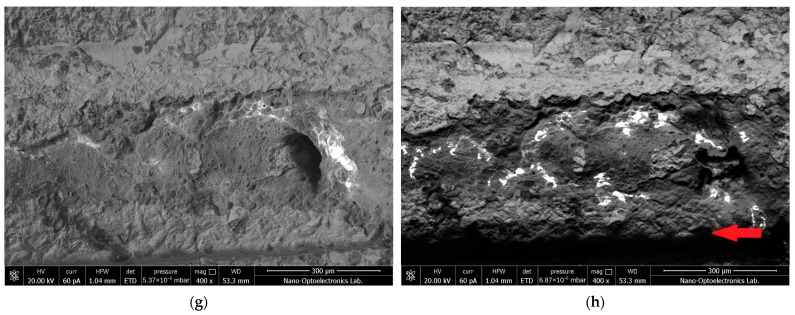
SEM images of ErCrL group implants before (T0) and after (T1) debridement, Specimen 9 (**a**,**b**,**e**,**f**); Specimen 10 (**c**,**d**,**g**,**h**). A 75× overview (**a**–**d**) with red boxes indicating the 400× (**e**–**h**) imaging regions; corresponding 400× detail. T0 (**a**,**c**,**e**,**g**) and T1 (**b**,**d**,**f**,**h**). Arrows indicate linear crack-like patterns across the treated surface.

Pre-debridement SEM images at 75× showed surface debris deposits over the threads and valleys in all five specimens of the ErCrL group (Figure 6a,c). Post-debridement images at 75× (Figure 6b,d) showed partial removal of deposits, with the residual debris forming a continuous layer rather than focal accumulations. In Specimen 10, this continuous layer showed a glossy, reflective appearance across the treated surface (Figure 6d). The appearance of linear crack-like patterns across the treated surface was observed in this treatment group (arrows in Figure 6b,d,f,h). At 400× magnification, the same regions showed dense debris deposits at T0 (Figure 6e,g), and the most contaminated valleys showed only a minimal reduction in debris layer thickness at T1 (Figure 6f,h).

#### 3.2.6. ErL Group

**Figure 7:**
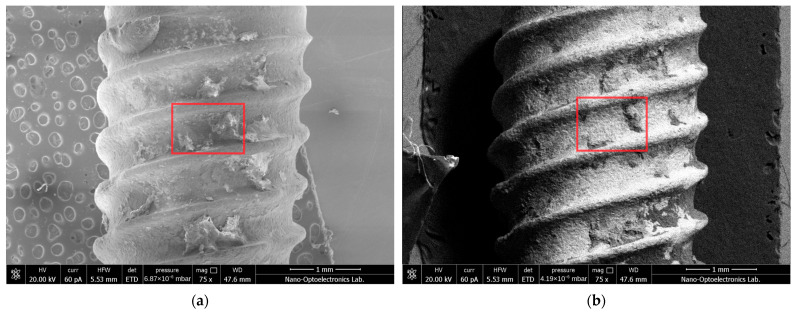

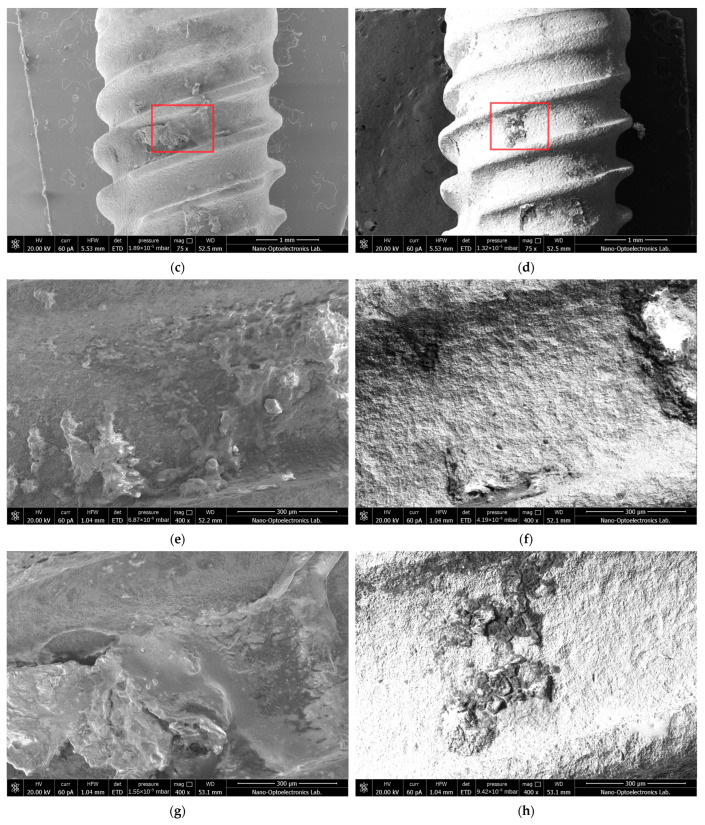
SEM images of ErL group implants before (T0) and after (T1) debridement, Specimen 11 (**a**,**b**,**e**,**f**); Specimen 12 (**c**,**d**,**g**,**h**). A 75× overview (**a**–**d**) with red boxes indicating the 400× (**e**–**h**) imaging regions; corresponding 400× detail. T0 (**a**,**c**,**e**,**g**) and T1 (**b**,**d**,**f**,**h**).

Pre-debridement SEM images at 75× show surface debris deposits over the threads and valleys in all five specimens of the ErL group (Figure 7a,c). Post-debridement images at 75× (Figure 7b,d) show partial debridement, with the debris layer becoming thinner but persisting on the surface. At 400× magnification, the same regions show surface debris deposits at T0 (Figure 7e,g). At T1, partial removal of the debris is observed (Figure 7f,h).

### 3.3. EDS Analysis

**Figure 8:**
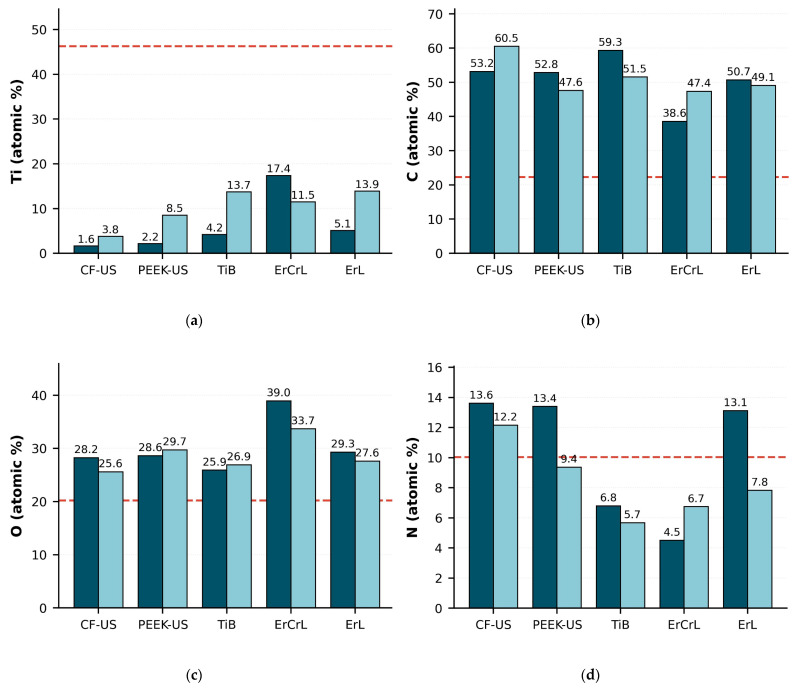
Atomic percentages of titanium (**a**), carbon (**b**), oxygen (**c**), and nitrogen (**d**) across all specimens in the five debridement groups before (T0) and after (T1) debridement, with pristine control shown as a red dashed line. The values above the bars are group medians. Ti: titanium; C: carbon; O: oxygen; N: nitrogen; CF-US: carbon fiber insert-ultrasonic; PEEK-US: PEEK insert-ultrasonic; TiB: titanium brush; ErCrL: Er,Cr:YSGG laser; ErL: Er:YAG laser; T0: pre-debridement (dark blue bars); T1: post-debridement (light blue bars).

All debridement groups at baseline (T0) had substantially lower titanium (median 1.65–17.39%) and substantially higher carbon (median 38.58–59.31%) than the pristine control (Figure 8). After debridement, compared to the control group, the at. % of carbon remained significantly higher and at. % of titanium remained significantly lower in all groups (respectively, *p* < 0.001, *p* = 0.023). Median atomic percentages of Ti, C, O, and N at T0 and T1, together with within-group changes (Δ = T1 − T0) and Wilcoxon signed-rank *p*-values, are provided in Table S1.

After debridement (T1), the direction of change in atomic carbon percentage differed across groups (Figure 8). Three groups showed a decrease: TiB (ΔC = −7.77 at. %), PEEK-US (ΔC = −5.24 at. %), and ErL (ΔC = −1.64 at. %). Two groups showed an increase: CF-US (ΔC = +7.35 at. %) and ErCrL (ΔC = +8.80 at. %). The atomic percentage of titanium increased in four of the five debridement groups post-debridement, approaching but not reaching the control level. The TiB group reached the highest post-debridement Ti value (13.72 at. %) and the CF-US group showed the lowest (3.77 at. %). The ErCrL group was the only group in which the atomic percentage of titanium decreased after debridement (ΔTi = −5.9 at. %). None of the debridement groups reached the control-level atomic profile for either Ti or C. The differences were not significant.

## 4. Discussion

In this study, SEM observations revealed clear morphological differences among the five debridement methods applied to peri-implantitis-failed SLA titanium implants. Overall, mechanical approaches achieved more extensive debris removal than laser-based methods. Among them, the rotating titanium brush provided the most effective cleaning, reaching thread–valley junctions and yielding the lowest residual contamination, together with the greatest reduction in surface carbon. Despite this effectiveness, neither SEM nor EDS findings indicated restoration of a pristine surface.

The two ultrasonic inserts demonstrated comparable debridement at low magnification but differed at higher resolution and in elemental outcomes. The carbon fiber insert produced substantial debris reduction (M-IDVI: 2.87 ± 0.87) but was associated with dark surface patches and localized flattening of the SLA microtopography. Its EDS profile showed increased surface carbon, likely reflecting deposition of carbonaceous wear debris, as previously described for reinforced polymer inserts [22]. In contrast, the PEEK insert debridement was limited (M-IDVI:2.93 ± 0.37) but resulted in a decrease in surface carbon, although residual material persisted within thread valleys. Prior studies similarly report similar results with PEEK, accompanied by limited polymer residue and minimal titanium particle release compared with metal inserts [18,23].

The laser systems showed different results both for cleaning efficacy and surface preservation. The Er:YAG laser preserved the SLA microtopography most closely but removed only a small proportion of debris (M-IDVI: 3.27 ± 0.83), as reflected by minimal changes in surface carbon. This aligns with previous findings indicating that, while Er:YAG can effectively remove biofilm, its ablation efficiency for more tenacious deposits is limited [24]. In contrast, the Er,Cr:YSGG laser produced more pronounced surface alterations, including a fused, glossy layer and microcrack patterns consistent with thermal modification. This group also exhibited increased surface carbon, a finding previously associated with laser-induced surface changes and possible retention or transformation of organic residues [16]. Differences between the two erbium systems may be explained by their wavelengths and interaction with water and substrate. The higher absorption of the Er:YAG wavelength supports more superficial and controlled ablation, whereas Er,Cr:YSGG may induce deeper thermal effects under similar conditions, particularly depending on tip design and energy delivery.

The titanium brush produced the most extensive debridement of any method (M-IDVI: 2.33 ± 0.33). The EDS profile showed the largest post-debridement reduction in surface carbon of any group. An early in vitro study reported that rotating titanium brushes reduce surface biofilm on rough titanium more effectively than steel curettes, while leaving the SLA microtopography apparently intact under SEM [25]. An XPS analysis comparing physical decontamination methods showed that titanium brushes were the most effective at removing organic contaminants from titanium discs, although none of the tested methods restored the pristine surface chemistry [26]. These previous reports are consistent with the present EDS observation that the TiB group did not recover the elemental profile of the pristine control.

A recent study using Er,Cr:YSGG with same device at 1.5 W/30 Hz with a side-firing tip on SLA titanium discs reported decontamination efficacy comparable to air-polishing without macroscopic disc damage at this energy setting [27]. SEM images in the present study, however, showed a distinctive linear crack pattern that was absent from the mechanical groups with high M-IDVI scores (3.73 ± 1.38). The EDS profile showed an increase in surface carbon after debridement, supported by another study of Er,Cr:YSGG irradiation at two different energy settings on peri-implantitis-failed implants [16]. Er,Cr:YSGG irradiation with a radial-firing tip at ≤1.5 W has been reported to produce extensive biofilm ablation on titanium discs without SEM-detectable damage [28]. Surface alteration also depends on tip and energy: conical tips at higher energies produce melting and cracking, while side-firing tips at lower energies do not [29].

The ErL showed less debris removal than mechanical methods (M-IDVI: 3.27 ± 0.83). The EDS profile showed the smallest post-debridement reduction in surface carbon content of any group. Despite this limited efficacy, the ErL group preserved the underlying SLA microtopography most closely out of any debridement group at 400× magnification. A comparative in vitro study on explanted SLA implants with naturally formed calculus demonstrated that Er:YAG achieved more effective calculus removal than cotton pellets or titanium curettes while preserving the microstructured surface; however, the same study noted that the ablation rate of calcified deposits is considerably slower than that of soft biofilm, requiring longer application times for complete removal [24]. A recent in vitro study identified 100 mJ at 10 Hz as the optimal energy for SLA titanium, achieving substantial biofilm removal while maintaining biocompatibility [30]. A previous in vitro study showed that the long-pulse mode of Er:YAG most effectively preserved the surface appearance, whereas the very-short-pulse mode used in the present study was associated with localized melting and microcracks at higher pulse energies [16].

A previous in vitro study from our group also used the explanted SLA implant model and the laser systems [16]. In the current study, we added state-of-the-art mechanical intervention methods as well as optimal recommended laser parameters. A previous study was designed based on a mildly contaminated implant. In the current study, we recruited implants with heavier debris on the surfaces, as evidenced by the SEM images at baseline. The use of heavily contaminated explanted implants represents a strength of this study, as it better reflects the complex surface characteristics of peri-implantitis-affected implants compared with flat disc models. Consistent with previous studies using similar models, complete restoration of the pristine surface was not achieved by any method. Additionally, by obtaining SEM and EDS measurements from the same pre-marked thread region before and after treatment, this study minimized variability and allowed direct assessment of treatment effects on a single surface. We modified our previous SEM visual index IDVI due to the inclusion of heavily contaminated implants. Thus, we were able to quantitatively measure residual debris. However, this index did not encompass surface damage. We also addressed this specifically.

The ErCrL group required the longest and the titanium brush required the shortest application time among the five groups. Unlike in our previous study, the treatment duration in the present study was not fixed. Instead, the procedure continued until the clinician visually confirmed complete debridement, as we believe the required application time is a critical factor in clinical practice. Among the techniques evaluated, the rotating titanium brush was found to be the fastest and most user-friendly, while providing comparable clinical outcomes.

Several limitations should be considered. As an in vitro study, the model does not replicate the influence of biological factors such as blood, saliva, and peri-implant soft tissues, which may affect both mechanical debridement dynamics and laser–tissue interactions. Surface chemistry was assessed only with EDS; complementary techniques such as X-ray photoelectron spectroscopy (XPS) would provide more detailed information about the titanium oxide layer and surface chemistry. Furthermore, the presence of heavier baseline contamination and the inclusion of polymer-based instruments may have influenced post-treatment elemental profiles, particularly carbon and oxygen levels. In addition, the explanted implants showed inherent baseline heterogeneity in contamination level and composition that could not be standardized. Microbiological and biological outcome measures, such as bacterial culture or cell-attachment assays, were not included in this study; these could complement the morphological and elemental assessments in future work.

## 5. Conclusions

SEM evaluation showed that mechanical debridement methods, particularly the rotating titanium brush, produced more extensive debris removal with minimal surface damage from peri-implantitis-failed SLA titanium implant surfaces. The Er,Cr:YSGG laser produced a melted surface layer, whereas the Er:YAG laser preserved the underlying SLA microtopography. No single contemporary debridement method fully restored the surface of a peri-implantitis-failed implant. Further research is required to evaluate a treatment protocol using Er:YAG lasers after thorough debridement with a rotating titanium brush.

## Figures and Tables

**Table 1:** Debridement protocol of each group.

Groups	Instruments	Parameters
CF-US (*n* = 5)	Ultrasonic scaler with carbon fiber insert (Periimpla Soft) ^1^	Maximum power setting (23 kHz); 30° angle; water cooling
PEEK-US (*n* = 5)	Ultrasonic scaler with PEEK insert (ICS + IC1) ^2^	Maximum power setting (36 kHz); 30° angle; water cooling
TiB (*n* = 5)	Rotating titanium brush (R-Brush) ^3^ on physiodispenser	4900 rpm; clockwise rotation; vertical motion; water cooling
ErCrL (*n* = 5)	Er,Cr:YSGG laser with RFPT5-14 radial firing tip ^4^	1.5 W; 30 Hz; 50 mJ/pulse; pulse duration 60 μs; air 60/water 50; energy density 19.23 J/cm^2^
ErL (*n* = 5)	Er:YAG laser with R02-C handpiece ^5^	VSP (100 μs); 100 mJ/pulse; 10 Hz; air 13/water 25; energy density 15.87 J/cm^2^
Control (*n* = 5)	Pristine implants; no intervention	—

CF-US: carbon fiber insert-ultrasonic; PEEK-US: PEEK insert-ultrasonic; TiB: titanium brush; ErCrL: erbium, chromium-doped yttrium scandium gallium garnet laser; ErL: erbium-doped yttrium aluminum garnet laser. Superscript numbers show the device used in each group: ^1^ Vector Paro (Dürr Dental, Bietigheim-Bissingen, Germany); ^2^ MyLunos Duo (Dürr Dental, Bietigheim-Bissingen, Germany); ^3^ R-Brush (NeoBiotech, Seoul, Republic of Korea); ^4^ WaterLase iPlus (Biolase, Foothill Ranch, CA, USA); ^5^ Fidelis Plus II (Fotona, Ljubljana, Slovenia).

**Table 2:** Distribution of implant dimensions by group.

Group	Length Median (mm)	Min–Max	Diameter Median (mm)	Min–Max
Control	12.0	8.0–12.0	3.8	3.5–4.3
CF-US	10.0	8.0–12.0	3.8	3.5–4.3
PEEK-US	10.0	8.0–12.0	3.8	3.5–4.3
TiB	10.0	8.0–12.0	3.8	3.5–4.3
ErCrL	10.0	8.0–12.0	3.8	3.5–4.3
ErL	10.0	6.0–12.0	3.8	3.5–4.3

CF-US: Carbon fiber insert-ultrasonic; PEEK-US: PEEK insert-ultrasonic; TiB: titanium brush; ErCrL: erbium, chromium-doped yttrium scandium gallium garnet laser; ErL: erbium-doped yttrium aluminum garnet laser. Kruskal–Wallis *H* test with Bonferroni correction for pairwise comparisons; *p* > 0.05 for all comparisons.

**Table 3:** Debridement time by group (seconds).

Group	Median (s)	Q1–Q3	Min	Max
CF-US	107	107–154	89	165
PEEK-US	124	106–153	91	166
TiB	74	65–75	52	75
ErCrL	332	304–356	124	420
ErL	114	85–116	63	118

CF-US: carbon fiber insert-ultrasonic; PEEK-US: PEEK insert-ultrasonic; TiB: titanium brush; ErCrL: erbium, chromium-doped yttrium scandium gallium garnet laser; ErL: erbium-doped yttrium aluminum garnet laser. Kruskal–Wallis *H* test with Bonferroni correction for pairwise comparisons; *p*-value > 0.05 for all groups.

**Table 4:** Post-treatment (T1) M-IDVI scores by group.

Sample	CF-US	PEEK-US	TiB	ErCrL	ErL
1	3/2/3	3/2/2	2/2/2	4/4/3	2/3/3
2	3/2/3	4/3/3	2/2/2	4/5/4	4/3/3
3	4/5/4	3/3/3	3/2/2	2/2/2	3/3/2
4	2/3/3	3/3/3	3/3/2	6/6/5	5/5/4
5	2/2/2	3/3/3	3/3/2	3/3/3	3/3/3
Mean ± SD	2.87 ± 0.87	2.93 ± 0.37	2.33 ± 0.33	3.73 ± 1.38	3.27 ± 0.83

CF-US: carbon fiber insert-ultrasonic; PEEK-US: PEEK insert-ultrasonic; TiB: titanium brush; ErCrL: erbium, chromium-doped yttrium scandium gallium garnet laser; ErL: erbium-doped yttrium aluminum garnet laser. Values are post-treatment (T1) M-IDVI scores from Observer 1, Observer 2, and Observer 3, respectively. The bottom row reports mean ± standard deviation of the five per-specimen mean scores within each group (*n* = 5 per group). Kruskal–Wallis H test with Bonferroni correction for pairwise comparisons; *p* > 0.05 for all comparisons. Inter-rater reliability among the three blinded observers: ICC(2,k) = 0.914.

## Data Availability

The data supporting the reported results are available from the corresponding author upon reasonable request.

## Supplementary Materials

The following supporting information can be downloaded at https://www.mdpi.com/article/10.3390/nano16120703/s1, Table S1: Surface elemental composition by EDS analysis: pre- and post-treatment atomic percentages with within-group change (*n* = 5 per group).

## Abbreviations

The following abbreviations are used in this manuscript:

ANOVA	Analysis of variance
AO/AAP	Academy of Osseointegration/American Academy of Periodontology
at. %	Atomic percentage
C	Carbon
CF-US	Carbon fiber insert-ultrasonic
EDS	Energy-dispersive X-ray spectroscopy
EFP	European Federation of Periodontology
Er,Cr:YSGG	Erbium, chromium-doped yttrium scandium gallium garnet
ErCrL	Er,Cr:YSGG laser
Er:YAG	Erbium-doped yttrium aluminum garnet
ErL	Er:YAG laser
ICC	Intraclass correlation coefficient
IDVI	Implant Debridement Visual Index
M-IDVI	Modified-Implant Debridement Visual Index
N	Nitrogen
O	Oxygen
PEEK	Polyetheretherketone
PEEK-US	PEEK insert-ultrasonic
SD	Standard deviation
SEM	Scanning electron microscopy
SLA	Sandblasted, large-grit, acid-etched
T0	Pre-debridement (baseline) time point
T1	Post-debridement time point
Ti	Titanium
TiB	Titanium brush
VSP	Very short pulse
XPS	X-ray photoelectron spectroscopy
