# Supplementary material for: Mechanical Versus Laser Debridement of SLA Titanium Implants: An In Vitro Morphological and Elemental Analysis of Debris Removal and Surface Preservation
Source: Nanomaterials (Basel). 2026 Jun 6;16(12):703. doi: 10.3390/nano16120703 (PMC13304573; doi:10.3390/nano16120703)
Supplement: Supplementary file 1 [file nanomaterials-16-00703-s001.zip › nanomaterials-4323189-supplementary.pdf]

Table S1. Surface elemental composition by EDS analysis: pre- and post-treatment atomic percentages with within-group change (n = 5 per group).

Group	Ti at%	C at%	O at%	N at%	p (Ti)	p (C)	p (O)	p (N)
**Control**	46.25 (46.00–46.61)	22.28 (21.64–22.56)	20.19 (20.06–20.37)	10.02 (9.98–11.26)	—	—	—	—
**CF-US**	1.65 (1.42–2.71) → 3.77 (3.19–5.52) *Δ +2.12*	53.16 (52.09–56.49) → 60.51 (56.14–61.41) *Δ +7.35*	28.24 (28.20–33.34) → 25.59 (24.09–27.43) *Δ -2.65*	13.62 (9.70–16.32) → 12.16 (8.13–14.22) *Δ -1.46*	0.893	**0.043***	0.080	0.500
**PEEK-US**	2.16 (2.15–7.90) → 8.53 (6.54–17.64) *Δ +6.37*	52.83 (51.95–54.45) → 47.59 (44.24–48.50) *Δ -5.24*	28.64 (28.54–32.69) → 29.72 (25.31–31.84) *Δ +1.08*	13.40 (10.74–14.95) → 9.37 (8.41–9.67) *Δ -4.03*	0.345	0.138	0.138	0.500
**TiB**	4.20 (3.53–9.59) → 13.72 (13.25–15.19) *Δ +9.52*	59.31 (44.88–62.22) → 51.54 (47.21–53.50) *Δ -7.77*	25.91 (22.80–33.08) → 26.90 (25.96–33.07) *Δ +0.99*	6.80 (5.18–6.85) → 5.67 (5.52–5.92) *Δ -1.13*	0.080	0.345	0.893	0.686
**ErCrL**	17.39 (8.86–20.13) → 11.49 (7.33–18.47) *Δ -5.90*	38.58 (34.53–39.06) → 47.38 (45.14–50.56) *Δ +8.80*	38.95 (34.90–39.37) → 33.67 (27.74–36.76) *Δ -5.28*	4.50 (4.44–6.03) → 6.74 (5.59–8.18) *Δ +2.24*	0.080	**0.043***	0.138	0.500
**ErL**	5.10 (2.18–5.89) → 13.88 (13.73–18.48) *Δ +8.78*	50.70 (50.07–51.86) → 49.06 (40.09–52.95) *Δ -1.64*	29.29 (26.82–31.40) → 27.61 (26.22–31.17) *Δ -1.68*	11.63 (9.64–13.12) → 7.83 (7.60–9.45) *Δ -3.80*	0.080	0.225	0.893	**0.043***

Element values are median (Q1–Q3) atomic percentages of titanium (*Ti*), carbon (*C*), oxygen (*O*), and nitrogen (*N*) at the implant surface, measured by energy-dispersive X-ray spectroscopy. Each element cell shows the median pre-treatment (**T0**) and post-treatment (**T1**) values with their interquartile ranges (Q1–Q3) in parentheses, and the within-group change in medians ($\Delta = T1 - T0$) on the second line. **CF-US**, carbon-fiber insert ultrasonic; **PEEK-US**, PEEK insert ultrasonic; **TiB**, titanium brush; **ErCrL**, Er,Cr:YSGG laser; **ErL**, Er:YAG laser. Pristine control implants were analyzed once (single time point). *p* values are within-group Wilcoxon signed-rank tests comparing T1 vs. T0 for each element; * $p < 0.05$.
